# A Systematic Review of Biological Control Agents, Plant Extracts and Cover Crops or Intercropping for the Control of *Leucoptera coffeella* (Lepidoptera: Lyonetiidae)

**DOI:** 10.3390/insects17060636

**Published:** 2026-06-16

**Authors:** Maguintontz Cedney Jean-Baptiste, Flávio Roberto Mello Garcia, Beatriz Sousa Coelho, Maria Aparecida Castellani, Mateus Pereira dos Santos, Aldenise Alves Moreira

**Affiliations:** 1 Programa de Pós-Graduação em Agronomia, Departamento de Fitotecnia e Zootecnia, Universidade Estadual do Sudoeste da Bahia, UESB, Vitória da Conquista 45083-900, BA, Brazil; 2025p0011@uesb.edu.br (M.C.J.-B.); 2022a0038@uesb.edu.br (B.S.C.); castellani@uesb.edu.br (M.A.C.); a.moreira@uesb.edu.br (A.A.M.); 2 Departamento de Ecologia, Zoologia e Genética, Instituto de Biologia, Universidade Federal de Pelotas, UFPel, Pelotas 96010-900, RS, Brazil

**Keywords:** control methods, entomopathogens, parasitoids, predators, natural enemies

## Abstract

The leaf miner *Leucoptera coffeella* Guérin-Méneville & Perrottet, 1842 (Lepidoptera: Lyonetiidae), is a major pest of coffee plants (*Coffea* spp.) worldwide. It is recognized for its widespread occurrence in coffee plantations and the quantitative and economic losses it causes to coffee production, especially in regions with higher temperatures and greater water deficits. We evaluated historical and current research approaches to leaf miner management, establishing current knowledge through a systematic review of research on biological control agents (BCAs), plant extracts and cover crops or intercropping for *L. coffeella* control, according to the Preferred Reporting Items for Systematic Reviews and Meta-Analyses (PRISMA) guidelines. We conducted a systematic review of research findings from 1980 to 2025, developing a set of a priori criteria for subsequent replication of the review process. This review covers 130 publications, of which 28 met the inclusion criteria. The selected studies were conducted in five countries, although 75.00% of the studies were from Brazil. The BCAs accounted for 60.71%, followed by plant extracts (32.17%) and cover crops or intercropping (7.15%). Field studies were predominant, prioritizing diagnostic studies, surveys, studies on alternative control methods, and finally studies on BCAs, providing an effective solution. The limitations and prospects for their management were analyzed, and we highlighted recommendations that will improve future studies.

## Simple Summary

*Leucoptera coffeella*, Guérin-Méneville & Perrottet, 1842 (Lepidoptera: Lyonetiidae), known as the coffee leaf miner, is among the insects that cause serious damage to coffee producers. It is a cosmopolitan pest found on coffee leaves in Africa, Asia and Neotropical countries, including Central America, the Caribbean islands and South America. This review explored historical and current research approaches for the management of *L. coffeella*, through a meta-analysis of research on biological control agents (BCAs), plant extracts and cover crops or intercropping for the control of *L. coffeella*. We reviewed research conducted worldwide on the biological control of the coffee leaf miner from 1980–2025. We examined 130 publications, developing a set of a priori criteria to verify how many focused on each approach, which control methods were used, and where the research was conducted. The selected studies were conducted in five countries, although 75.00% of the studies were from Brazil. The BCAs accounted for 60.71% of the studies, followed by plant extracts at 32.14% and cover crops or intercropping at 7.15%. Regarding methodological approaches, 16 publications were field studies, eight laboratory studies, three combined studies, and one a greenhouse study. Studies under field conditions were more predominant, prioritizing diagnosis, surveys, and control alternatives, followed by biological control through BCAs, describing an effective alternative for sustainable and resilient production systems.

## 1. Introduction

Coffee cultivation has a fundamental role in generating employment and income, being especially present in family farming [1]. It is one of the most traded commodities in the world, with Brazil being the largest producer and exporter of the beans, representing 38% and 66.4 million tons (MT). Vietnam (17%; 30.1 MT), Colombia (7%; 12.9 MT), Indonesia (6%; 10.9 MT), Ethiopia (5%; 8.36 MT), Uganda (4%; 6.5 MT), India (4%; 6.2 MT), Honduras (3%; 5.3 MT), Peru (2%; 4.35 MT), and Mexico (2%; 3.87 MT) also contribute to world coffee production [1,2].

Among the phytosanitary problems related to this crop, attack by the coffee leaf miner, *Leucoptera coffeella,* Guérin-Méneville & Perrottet, 1842 (Lepidoptera: Lyonetiidae), stands out, especially in South America, Central America, and the Caribbean [3,4,5,6]. *Leucoptera coffeella* is a pest that causes significant damage to coffee plantations, since the larvae feed on the parenchyma, reducing photosynthetic rates and significantly decreasing coffee productivity [6,7].

Several methods exist for managing this pest, including application of chemicals and semiochemicals; genetic, botanical, and classical breeding approaches; and release of entomopathogenic fungi (EPFs), bacteria, and natural enemies like predators and parasitoids [6,8,9,10]. While often limited in efficacy and sustainability, biological alternatives can be effective [11]. Furthermore, Integrated Pest Management (IPM) offers a sustainable alternative to conventional chemical control by enhancing natural enemy populations [12].

The EPFs have been widely used as effective biological control agents for arthropods, such as those of the class Arachnida, frequently causing epizootics and significantly reducing host populations [13]. Fungal infection occurs through contact, directly through penetration of the cuticle, exerting multiple mechanisms of action and conferring a strong capacity to prevent the host from developing resistance [14].

The EPFs *Beauveria bassiana* (Bals.) Vuill., *B. brongniartii* (Sacc.) Petch., *Metarhizium anisopliae* (Metschn.) Sorokin, and *Isaria fumosorosea* Wize are widely used for biological pest control [15]. The fungus *B. bassiana* is a promising agent against arthropods due to its wide host range, easy dispersal, and ability to infect via the cuticle, respiratory tract, and digestive tract [16,17,18]. *Metarhizium anisopliae* infects hosts by penetrating the cuticle, beginning with conidia adherence through hydrophobic interactions [19].

Braconidae and Eulophidae wasp species also help regulate coffee pest populations [20,21]. Of 28 known coffee leaf miner parasitoids worldwide, 13 are present in Brazil, primarily from these families [22,23]. Moreover, among the predators of the Vespidae family identified as biological control agents of *L. coffeella* [24], at least 11 species have been recorded in Brazil [25].

Botanical pesticides provide effective, safe, and environmentally friendly alternatives to synthetic chemicals [26]. Natural pesticides (plant extracts/oils) are internationally recognized for their effective and stainable pest control [27]. Studies have shown that extracts from *Coffea racemosa* Lour, neem seed extract (NeemAz T/S), and orange oils can reduce *L. coffeella* oviposition and larval hatching [28,29].

This review analyzes current knowledge, limitations, and key factors driving the success of biological control, botanical pesticide application, and cover cropping/intercropping for *L. coffeella*. The following questions are addressed: (1) What is the most studied group of biological control agents (BCAs) that contribute to the control of *L. coffeella*? (2) What are the most common methodological approaches used by researchers? (3) What is the commonly adopted research scope? (4) How were the success and failure of control determined in studies?

## 2. Materials and Methods

### 2.1. Search Criteria

We used Scopus, Elsevier, PubMed, Google Scholar, and SciELO to generate a database of publications that have evaluated the control of *L. coffeella* in a pest management context. The search terms entered were: Coffee leaf miner OR “*Leucoptera coffeella*” OR “biological control” OR “natural enemies” OR “parasitoids” OR “predators” OR “bacteria” OR “fungi” OR “biopesticides” OR “botanical pesticides” OR “bioactive” OR “plant extracts” OR “survey” OR “management” OR “diagnosis” OR “control” OR “alternative controls“. To ensure scientific rigor, this study was limited to complete research articles that had undergone a peer-review process. The publications were written in Spanish, Portuguese, and English and published in journals between 1980 and 2025.

### 2.2. Data Inclusion and Exclusion Criteria

**Figure 1:**
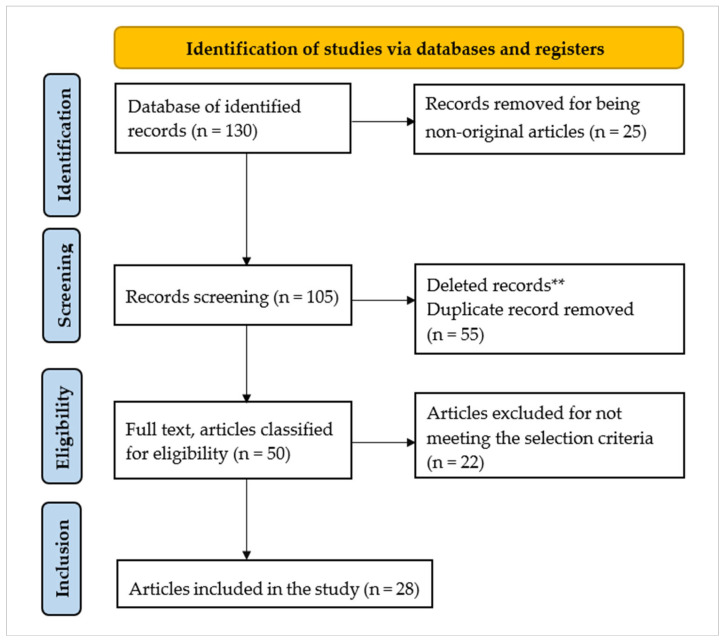
PRISMA 2020 flow diagram. Illustrating the search strategy for selecting articles for systematic review on the use of BCAs, plant extracts and cover crops/intercropping for the management of *Leucoptera coffeella.* ** Records excluded because they were present in multiple databases.

We obtained 130 initial results. For study inclusion, the following criteria were used: (1) studies of the species *L. coffeella*; (2) experiments on the biological control of *L. coffeella* by at least one parasitoid, predator, or entomopathogen; and (3) experiments involving at least one plant extract and/or botanical pesticides and studies of intercropped coffee plantation systems/cover crops. For exclusion, some publications were removed according to the following criteria: (1) studies without focus on control strategies, management, alternative controls, and biological controls; (2) review articles and conference proceedings; (3) books and book chapters; (4) editorials of scientific notes, dissertations, and theses; (5) studies characterizing *L. coffeella*, identifying *L. coffeella*, insecticide selectivity, studies without obvious links to BCAs, botanical extracts, and *L. coffeella* alone. We followed PRISMA guidelines (Supplementary Material File S1); http://www.prisma-statement.org (accessed on 6 June 2025) [30,31] (PRISMA 2020 statement and checklist) to include or exclude publications during the screening steps. A systematic review checklist is presented in the Supplementary Material File S2. The inclusion and exclusion process is shown in Figure 1.

### 2.3. Data Extraction and Processing

For data extraction, Citavi 6.3.0.0 software [32] and Swiss Academic Software, 2019 were used to import and classify the included studies. For each publication, the complete reference was collected and information was extracted on the variables of interest such as (1) pest; (2) country where the study was developed; (3) host; (4) subject of the work; (5) BCAs, plant extracts/botanical extracts; (6) methodological approaches used in each study (laboratory, field, greenhouse, and combined laboratory + greenhouse); (7) % parasitism, % predation, % mortality, exposed stage of *L. coffeella*, experiment duration, efficacy approaches; (8) studies including trials with intercropped coffee plantations/cover crop systems.

### 2.4. Statistical Analysis

The results of the eligible studies are characterized and presented in graphs and tables. Data about years of publications, countries and number of publications, methodological approaches and control methods, and publications evaluating BCAs are detailed and generated in graphs using the Bio-Stat software version 5.3 [33].

## 3. Results

### 3.1. Years of Publications

**Figure 2:**
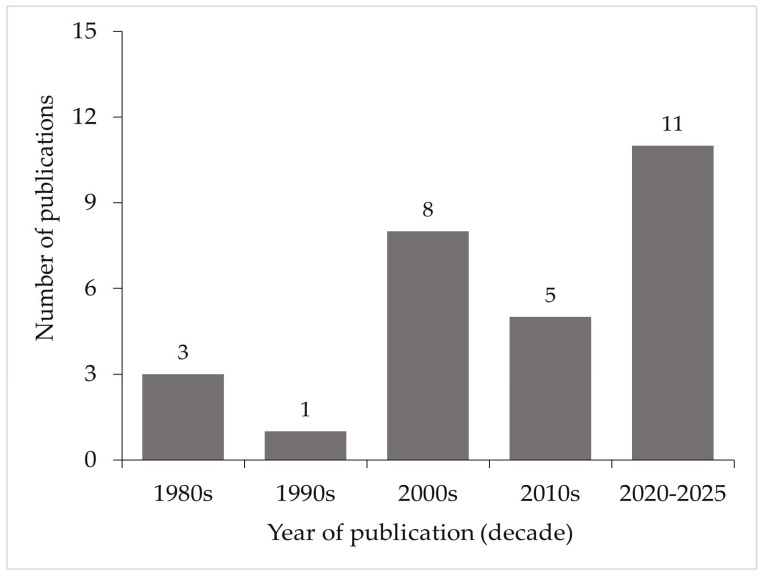
Temporal trend of BCAs, plant extracts, and cover crops/intercropping for the management of *Leucoptera coffeella.*

One hundred and thirty publications were found, of which 25 were removed. Analysis of 105 of these publications guided the selection of 50, but full reading led to the exclusion of 22 more of them for not meeting the proposed criteria (Figure 1). The complete references of all publications and the extracted data are presented in Supplementary Material File S2. The systematic review flowchart contains the steps for identification, screening, assessing eligibility and including articles, along with their respective quantities (Figure 1). An increase in research was noticeable from the 2000s (28.57%, n = 8), demonstrating expansion of scientific interest in *L. coffeella.* Moreover, from 2010 to 2019, the studies decreased by 17.86% (n = 5) (Figure 2).

### 3.2. Countries and Number of Publications

**Figure 3:**
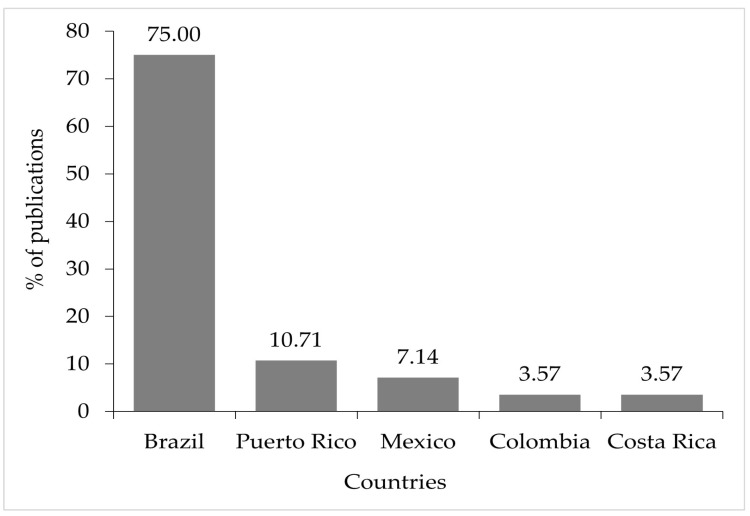
Number of publications per country on BCAs, plant extracts, and cover crops/intercropping involving *Leucoptera coffeella*.

The research studies were conducted in five countries (Figure 3), with 75.00% (n = 21) in Brazil, 3.57% (n = 1) in Colombia, 7.14% (n = 2) in Mexico, 10.71% (n = 3) in Puerto Rico, and 3.57% (n = 1) in Costa Rica. Publications from these countries included diagnostic studies, surveys, studies of management, alternative controls, and BCAs (Supplementary Material File S2) (Figure 3, Table 1, Table 2, Table 3 and Table 4).

**Table 1:** Characteristics and results of entomopathogens (entomopathogenic fungi) and cover crops/intercropping.

Country	Species	Experimental Sites	Results	References
**Brazil**	*Metarhizium anisopliae* (Metschn.) Sorokin (Clavicipitaceae)	Laboratory	The CM-14 isolate of the fungus *M. anisopliae* demonstrated high virulence against *L. coffeella* eggs, showing in 100% larval mortality in spore concentrations of 0.0019 and 0.031 g/mL.	[34]
**Brazil**	*Isaria fumosorosea* Wize (Cordycipitaceae)	Field	*Isaria fumosorosea* showed mortality rates ranging from 55.19 to 62.34% compared to *L. coffeella.*	[35]
**Brazil**	Coffee plants × banana trees	Field	There was a positive and significant relationship between increased plant diversity and wasp predation of coffee plantations in the shadeless system.	[36]
	Coffee vs. pigeon pea			
**Brazil**	*Crotalaria juncea* L. (Fabaceae)	Field	An increase in the parasitism rate of *L. coffeella* was observed in coffee intercropped with *F. esculentum.*	[37]
	*Fagopyrum esculentum* Moench (Polygonaceae)			

### 3.3. Methodological Approaches and Control Methods

**Figure 4:**
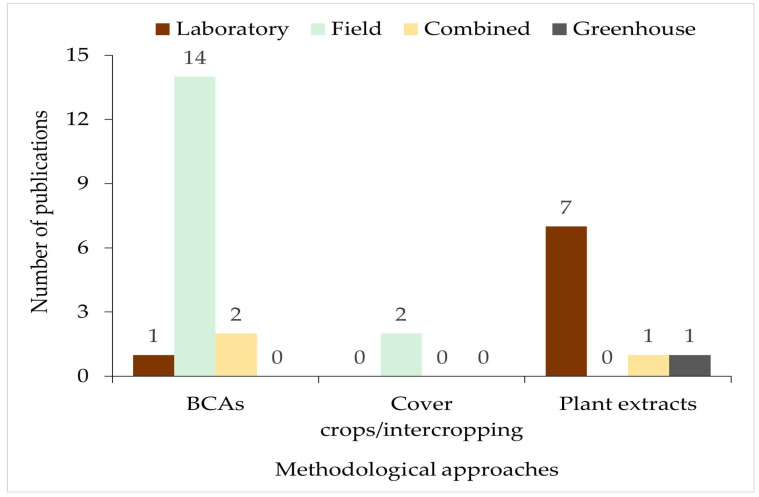
Methodological approaches applied in the publications on BCAs, cover cropping/intercropping and plant extracts of the coffee leaf miner, *Leucoptera coffeella.* Combined: (laboratory + greenhouse); Field; Laboratory; Greenhouse.

The BCAs were the most represented (60.71%, n = 17), followed by botanical extracts (32.14%, n = 9) and cover cropping/intercropping (7.15%, n = 2) (Figure 4). Regarding methodological approaches, 16 publications (57.14%) were field studies, 28.57% (n = 8) were laboratory studies, 10.71% (n = 3) were combined studies, and 3.57% were greenhouse studies (n = 1) (Figure 4, Table 1, Table 2, Table 3 and Table 4).

### 3.4. Publications Evaluating BCAs

**Figure 5:**
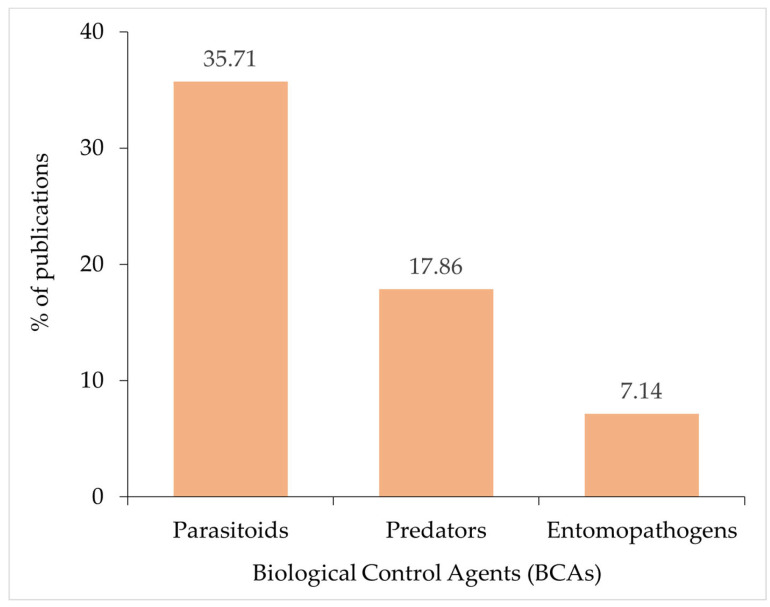
Comparison between parasitoids, predators and entomopathogens (entomopathogenic fungi) BCAs of *Leucoptera coffeella*.

The studies were classified into approaches such as biological control, alternative control, survey, diagnostic, and management (Supplementary Material File S2). Seventeen studies (60.71%) were considered eligible for the BCAs group, with 35.71% (n = 10) focusing on parasitoids, 17.86% (n = 5) on predators, and 7.14% (n = 2) on entomopathogens (Figure 5, Table 1, Table 2 and Table 3).

The studies analyzed referred to control interventions implemented in five countries and published from 1983 to 2025. Twenty-eight studies were considered eligible for this systematic review and description of their characteristics. For each study, we present in tabular form the results of methodological approaches such as BCAs (parasitoids, predators or and entomopathogens), plant extracts and cover cropping/intercropping (Table 1, Table 2, Table 3 and Table 4).

**Table 2:** Characteristics and results of parasitoids.

Country	Species	Experimental Sites	Results	References
	Hymenoptera–Eulophidae			
**Brazil**	*Cirrospilus neotropicus* Diez & Fidalgo, 2003	Field	*Neochrysocharis coffeae* was classified as constant and dominant and considered the only predominant species, presenting a higher frequency than the other species.	[21]
	*Closterocerus cofeellae* Ihering, 1914			
	*Horismenus aeneicollis* Ashmead, 1904			
	*Neochrysocharis coffeae* Ihering, 1914			
	*Stiropius* sp.1			
	*Stiropius* sp.2			
**Brazil**	*Proacrias coffeae* Ihering, 1914	Field	The larval parasitoids *P. coffeae* and *C. coffeellae* of *L. coffeella* were the most harmful as natural control agents. Their effectiveness varies according to environmental conditions.	[38]
	*Zagrammosoma* sp.			
	*Closterocerus coffeellae* Ihering, 1914			
	*Closterocerus flavicinctus* De santis, 1980			
	Hymenoptera–Eulophidae			
**Brazil**	*Horismenus* sp.	Field	The total mortality levels of eggs, larvae and pupae of *L. coffeella* were 38.5%, 43.8%, and 12.7%, respectively. *C. striata* was more efficient, accounting for 62.8% of pupal mortality caused by parasitism.	[3]
	*Cirrospilus* sp.			
	*Proacrias coffeae* Ihering, 1914			
	*Closterocerus coffeellae* Ihering, 1914			
	Hymenoptera–Braconidae			
	*Orgilus niger* Penteado-Dias, 1999			
	*Centistidea striata* Penteado-Dias, 1999			
	Hymenoptera–Braconidae			
**Mexico**	*Allobracon* spp.	Field	*Neochrysocharis* spp., *S. letifer*, *Pnigalio* spp. and *Z. multilineatum* were recovered most frequently. They are larval parasitoids, of which 78%, 18 species or morphospecies, were recovered from leaf miners, although some emerged from coffee leaf miner larvae within cocoons, which represented 22% or four species or morphospecies.	[39]
	*Styropius letifer* (Mann)			
	Hymenoptera–Eulophidae			
	*Cirrospilus* spp.			
	*Closterocerus* spp.			
	*Closterocerus cinctipennis* Ashmead, 1888			
	*Elachertus* spp.			
	*Horismenus* spp.			
	*Myotropis* sp.			
	*Neochrysocharis* spp.			
	*N. arvensis* Graham, 1963			
	*N. chalybea* Hansson, 1995			
	*N. formosa* Westwood, 1833			
	*Pnigalio* spp.			
	*P. sarasolai* De Santis, 1983			
	*Zagrammosoma lineaticeps* Girault, 1915			
	*Z. multilineatum* Ashmead, 1888			
	Hymenoptera–Eulophidae	Field	In two years of survey work (1985–1986), the authors identified parasitoids of *L. coffeella* and reported that the average parasitism rate was 23.5%, with the highest levels of parasitism being 66.7% in 1985; and 54.0% in 1986.	[40]
**Puerto Rico**	*Cirrospiloideus* sp.			
	*Zagrammosoma* sp.			
	*Horismenus* sp.			
	*Achrysocharoides* sp.			
	*Chrysonotomyia* sp.			
	Hymenoptera–Braconidae			
	*Mirax insularis* Muesebeck, 1937			
	Hymenoptera–Braconidae			
**Brazil**	*Stiropius reticulatus* Penteado-Dias, 1999	Field	Overall, the abundance of braconid specimens (67.6%) was higher, but species richness was higher in eulophids. The braconids *O. niger* and *S. reticulatus* were well adapted as *L. coffeella* control providers, with a higher level of parasitism compared to the other parasitoids.	[22]
	*Orgilus niger* Penteado-Dias, 1999			
	*Centistidea striata* Penteado-Dias, 1999			
	*Cirrospilus neotropicus* Diez and Fidalgo, 2004			
	Hymenoptera–Eulophidae			
	*Cirrospilus* sp.			
	*Closterocerus coffeellae* Ihering, 1914			
	*Horismenus aeneicollis* Ashmead, 1904			
	*Proacrias coffeae* Ihering, 1914			
	Hymenoptera–Braconidae			
**Puerto Rico**	*Mirax insularis* Muesebeck, 1937	Field	The braconid *M. insularis* was introduced to Puerto Rico for a biological control program aimed at regulating *L. coffeella* populations, showing promise due to its habitat stability, *L. coffeella* specificity, indirect pest control and environmental preservation.	[41]
	Hymenoptera–Braconidae			
**Puerto Rico**	*Mirax insularis* Muesebeck, 1937	Field	The braconid *M. insularis* is a koinobiont with a life cycle of 15 to 17 days, very similar to that of *L. coffeella* and well distributed in all ecological zones of the coffee-growing region of Puerto Rico, exerting a parasitism level of 14.8% on *L. coffeella.*	[42]
	Hymenoptera–Eulophidae			
**Colombia**	*Zagrammosoma multilineatum* Ashmead, 1888	Field	*Leucoptera coffeella* infestation rate of less than 2% and parasitism levels between 58 and 89%. Of the seven species collected, *Horismenus* n. sp. and *Apleurotropis* n. sp. were classified as new to Colombia, but *C. coffeella* was effective in controlling *L. coffeella*.	[43]
	*Pnigalio sarasolai* De Santis, 1983			
	*Closterocerus coffeellae* Ihering, 1914			
	*C. lividus* Ashmead, 1886			
	*Horismenus* sp.			
	*Horismenus* n. sp.			
	*Apleurotropis* n. sp.			
	Hymenoptera–Eulophidae			
**Brazil**	*Closterocerus coffeellae* Ihering, 1914	Field	In areas with more trees and adjacent vegetation, with less pesticide use, there was an increase in the quantity and variety of natural enemies of *L. coffeella*, which helps in more effective pest control.	[44]
	*Proacrias coffeae* Ihering, 1914			
	*Stiropius reticulatus* Penteado-Dias, 1999			
	*Neochrysocharis* sp. 1			
	*Neochrysocharis* sp. 2			
	*Zagrammosoma* sp.			

**Table 3:** Characteristics and results of predators.

Country	Species	Experimental Sites	Results	References
	Neuroptera–Chrysopidae			
**Brazil**	*Chrysoperla externa* Hagen, 1861	Combined	The average daily attack rate (a’) for *Ch. externa*, *Ce. cincta* and *Ce. cornuta* was 0.009 for both larvae and pupae. The average handling times (T/h) were 3.5 h and 3.7 h for *L. coffeella* larvae and pupae, respectively. The average estimated number of prey attacked (24 h/Th) during the 24 h observation period was 6.9 and 6.6 for *L. coffeella* larvae and pupae, respectively.	[45]
	*Ceraeochrysa cincta* Shneider, 1851			
	*Ce. cornuta* Navás, 1925			
**Brazil**	*Chrysoperla externa* Hagen, 1861	Field	*Leucoptera coffeella* lesions on the trees ranged from 45 to 69% in the lace release treatment and from 43 to 67% in the organic management treatment.	[46]
**Brazil**	*Ceraeochrysa cubana* Hagen, 1914	Field	The presence of first, second, and third instar *C. cubana* larvae reduced the percentage of mine formation by 70.0, 73.6 and 53.3%, respectively.	[8]
	Hymenoptera–Vespidae–Formicidae			
**Brazil**	*Polybia scutellaris* White, 1841	Combined	They break through the leaf’s epidermis, both on the upper and lower surfaces, to access and prey on the leaf miner larva.	[23]
	*Brachygastra lecheguana* Latreille, 1824			
	*Protonectarina sylveirae* Saussure, 1854			
**Mexico**	*Azteca instabilis* Fr. Smith, 1862	Field	The presence of *A. instabilis* did not influence the presence or severity of damage caused by *L. coffeella*. The vegetation cover around the trees ranged from 2.5% to 93.5%. The number of trees per plot ranged from 6 to 29 and the tree species richness from 3 to 11.	[24]

**Table 4:** Characteristics and results of commercial botanical insecticides, essential oils and other botanical extracts.

Country	Plant Species	Experimental Sites	Results	References
Brazil	*Plantago lanceolata* L. (Plantaginaceae)	Combined	Extracts of *P. lanceolata* and *M. charantia* on oviposition preference and development of *L. coffeella* under laboratory and/or greenhouse conditions reduce oviposition and egg hatching, apparently as a result of the action of plant metabolites on the embryo.	[26]
	*Momordica charantia* L. (Cucurbitaceae)			
**Brazil**	Orange oil	Laboratory	The orange oil reduced the formation of mines on coffee leaves by 46.8%. The botanical insecticide neem prevented insects from completing the pupal stage and orange essential oil reduced pre-adult survival by 37.7% and their lifespan by 2.7 days.	[10]
	Neem oil			
**Brazil**	*Ruta graveolens L.*(Rutaceae)	Laboratory	Aqueous extracts of *T. ciliata* were the most efficient, reducing the number of *L. coffeella* eggs on coffee leaves. Extracts of *T. casaretti* and *T. pallida* also reduced the oviposition rate. Coffee leaves treated with *A. indica* (S) showed the lowest percentage of mined area/larva.	[47]
	*Melia azedarach* L. (Meliaceae)			
	*Toona ciliata* M. Roem (Meliaceae)			
	*Trichilia casaretti* C. DC. (Meliaceae)			
	*T. pallida* SW. (Meliaceae)			
	*T. catigua* A. Juss (Meliaceae)			
	*Azadirachta indica* A. Juss (Meliaceae)			
	*Chenopodium ambrosioides* (L.) Mosyakin & Clemants (Amaranthaceae)			
**Brazil**	Pirolenhoso Biopirol ^®^	Laboratory	Pyroligneous Biopirol^®^ extract at concentrations (2, 4, 8 and 16%) and Azadirachtin Nim-I-Go^®^ at concentrations (0.25, 0.5, 0.75 and 1%) tested in the laboratory were slightly toxic to *L. coffeella* and do not show toxic effects in the field.	[48]
	Azadirachtin Nim-I-Go ^®^			
**Costa Rica**	Emulsifiable neem oil	Laboratory	*Lecoptera coffeella* eggs deposited on leaves treated with emulsifiable oil concentrations 0.125, 0.25, 1.25 and 2.5% showed an average oviposition rate of 52.3% at the concentration 0.125% and average oviposition rate of 61.6% at the concentration of 2.5%.	[49]
**Brazil**	Brown propolis	Laboratory	Neem oil at the concentration 1% had mortality rates of 98.4% and 81.6% in *L. coffeella* caterpillars in the first and third larval stages, respectively.	[50]
	Pyroligneous pepper and garlic			
	Neem oil			
**Brazil**	*Handroanthus impetiginosus* (Mart. Ex DC.) Mattos (Bignoniaceae)	Laboratory	The seed extract at the concentration 1% showed the highest efficacy, reducing egg hatching by 63%, preventing pupal development, and completely inhibiting adult oviposition. Application to eggs, pupae, and adults resulted in a significant reduction in the pest’s survival rate and, in some cases, completely prevented insect development (100% mortality).	[51]
**Brazil**	*Citrus limon* (L.) Osbeck (Rutaceae)	Laboratory	Freeze-dried extracts of *G. hederacea* (44.8%), *M. sylvestris* (62.7%), *M. jalapa* (49.2%)*, P. Alliaceae* (58.7%), *P. ruderale* (42.4%), *R. officinalis* (27.9%)*, S. nigra* (35.8%) and *T. majus* (44.4%), *M. indica* (27.4%), *M. sapientum* (37.7%), *O. basiculum* (43.2%), and *P. guajava* (23.5%) increased mortality of *L. coffeella* larvae. *Ocimum basiculum* (43.2%), *C. limon* (38.6%), *M. sylvestris* (62.7%), *M. jalapa* (49.2%), *G. hederacea* (44.8%), and *T. majus* (44.2%) caused the highest mortality rates.	[52]
	*Glechoma hederacea* L. (Limaceae)			
	*Malva sylvestris* L. (Malvaceae)			
	*Mangifera indica* L. (Anacardiaceae)			
	*Mirabilis jalapa* L. (Nyctaginaceae)			
	*Musa sapientum* L. (Musaceae)			
	*Ocimum basiculum* L. (Lamiaceae)			
	*Petiveria alliaceae* B.A. Gomes (Phytolaccaceae)			
	*Porophyllum ruderale* (Jacq.) Cass. (Asteraceae)			
	*Psidium guajava* L. (Myrtaceae)			
	*Rosmarinus officinalis* L. (Lamiaceae)			
	*Sambucus nigra* L. (Adoxaceae)			
	*Tropaeolum majus* L. (Tropaeolaceae)			
**Brazil**	NeemAzal T/S	Greenhouse	NeemAzal T/S showed its effect on the oviposition of *L. coffeella* in a greenhouse, reducing oviposition by an average of 36.77%. Female *L. coffeella* oviposited on coffee plants treated with 0.1 g/L of azadirachtin, but miner development was interrupted when leaves containing *L. coffeella* eggs or larvae were treated with 0.0251 g/L or 0.1 g/L of azadirachtin.	[27]

## 4. Discussion

### 4.1. Fungal Products

Biological control with EPFs has been used as an alternative to chemical products for a wide range of insect pests [53]. *Isaria fumosorosea*, when applied alone, constitutes a viable tool for the management of *L. coffeella* [35]. Its action on leaf miner larvae is explained by the contact mechanism: spores deposited on the leaf surface reach newly hatched specimens prior to mine formation [35,54]. *Leucoptera coffeella* in all its biological states is highly susceptible to infection by *M. anisopliae*, isolate CM-14 [34,35,36,37,38,39,40,41,42,43,44,45,46,47,48,49,50,51,52,53,54,55]. The fungus, by penetrating the egg chorion, acts as an entomopathogenic inoculum for newly hatched larvae. As a saprophytic fungus, it develops in the excrement deposited in the egg chorion, from where the larva penetrates the leaf, invading the insect’s gallery and thus reaching the host. In this context, larvae that do not die within the gallery may be killed by *M. anisopliae* when they bore into it in search of a pupation site. The spores on the leaf surface come into contact with the larvae and will kill them in the pre-pupal stage [34].

### 4.2. Parasitoids

Eulophids and braconids are the most frequently observed parasitoids in coffee leaf miners, as they are more effective BCAs, contributing to the regulation of the pest population in coffee plantations [39]. Studies conducted in South America, Central America, and the Caribbean have confirmed the presence of parasitoids in galleries in coffee leaves with miners of *L. coffeella*. In Brazil, some researchers have reported the role of natural enemies of *L. coffeella* in several states, such as Paraná, São Paulo, Minas Gerais, and Bahia, including *C. neotropicus*, *C. coffeellae*, *H. aeneicollis*, *N. coffeae*, *Stiropius* sp. 1, and *Stiropius* sp. 2 of the Eulophidae family associated with *L. coffeella* [21].

The effectiveness of the parasitoid community, its specificity, and its tolerance to *L. coffeella* vary among growing regions due to biotic and abiotic factors in the coffee agroecosystem and population dynamics of *L. coffeella* [56]. The primary parasitoids belong to the genera *Neochrysocharis*, *Pnigalio* and *Zagrammosoma*, *Stiropius* (Hymenoptera: Eulophidae). These parasites may appear more frequently in *L. coffeella* larvae inside cocoons [39]. In the 1970s, in Puerto Rico the parasitoids *Cirrospiloideus* sp., *Zagrammosoma* sp., *Horismenus* sp., *Achrysocharoides* sp., and *Chrysonotomyia* sp. of the Eulophidae family were widely reported in coffee plantations as natural mortality factors, as was an exotic braconoid, *M. insularis* [40,41,42,43], a koinobiont with a life cycle ranging from 15 to 17 days, which was introduced to suppress *L. coffeella* populations [42]. In Colombia, *C. coffeellae* has been recommended for the management of *L. coffeella* through a conservationist biological control strategy [43].

The diversity of *L. coffeella* parasitoids is favored by low insecticide use, high leaf density, and proximity to forest areas [22]. Thus, shaded or transitional biological systems, as well as sunny, pesticide-free plantations, are conducive to the preservation of these hymenopterans [38]. The population dynamics of *L. coffeella* can be strongly affected not only by the characteristics of the host plant and environmental conditions, but also by the abundance of natural enemies [3,6]. Research on the occurrence of the species *P. coffeae*, *Zagrammosoma* sp., ***C.*** *coffeellae*, *C. flavicinctus* (Hymenoptera: Eulophidae), related to *L. coffeella* in Brazil was carried out by Gravena (1983) in the municipality of Jaboticabal, SP, showing that the activity of *L. coffeella* can increase with decreasing temperature and relative humidity, and that the number of live larvae of this species can be influenced by these factors and precipitation [38,44].

### 4.3. Predators

In coffee-producing regions of Brazil, *L. coffeella* is frequently found in areas without shade that are dry and hot [57]. Several predators have already been identified as BCAs of *L. coffeella.* Lacewings, wasps, ants, and thrips are known predators in the field that exhibit greater or lesser effectiveness in predation, depending on the species [8,24,45]. Ants are social insects that can destroy the galleries of *L. coffeella* to feed on their larvae. The species *B. lecheguana*, *P. scutellaris* and *P. occidentalis* remove the larvae from the underside of the miners and the species *P. silveirae* from the top [24].

*Ceraeochrysa cubana* is known as a generalist predator of agricultural pests, and it has recently been discovered that it successfully attacks *L. coffeella* eggs and third-instar larvae after they enter the galleries. Their first-instar larvae can feed on *L. coffeella* eggs immediately after hatching [8].

The release of *Ch. externa* combined with the application of orange and neem essential oils reduces *L. coffeella* populations in coffee plantations, by decreasing the number of larvae and pupae [46]. The species *Ch. externa*, *Ce. cincta*, and *Ce. cornuta* studied under laboratory conditions exhibited a functional response to increased densities of *L. coffeella* larvae and pupae. This indicates that the daily consumption of these predators increases proportionally to the amount of prey available (*L. coffeella* larvae or pupae), until it reaches a stabilization point at high population densities [45].

### 4.4. Intercropping Plants/Cover Plants

By diversifying vegetation, plant intercropping can increase the presence of predatory wasps, and consequently reduce the population of *L. coffeella*. Furthermore, the interactions between morphological and ecological characteristics of plants, herbivores, and natural enemies can be a critical factor for the development of functional diversity [36]. Diversifying coffee plantations with cover crops can be a strategy able to increase the availability of plant food for natural enemies, improving their survival and resulting in greater effectiveness of pest control [36,37]. For example, the effect of combining *C. juncea* and *F. esculentum* can ensure an increase in the parasitism rate of *L. coffeella* by mites of the family Phytoseiidae [37].

### 4.5. Commercial Botanical Insecticides—Essential Oils

Plant extracts and phytoprotective agents with insecticidal properties are also promising alternatives for the control of *L. coffeella* [49]. In the 1980s, many researchers attributed their interest in botanical insecticides to neem, *A. indica*, and particularly to its commercial development in the United States and Germany, as it constitutes an important source of studies on insecticidal properties [26]. In Brazil, *A. indica* is the most studied plant and there is much research on its insecticidal activity against various pest insect species, such as *L. coffeella*, the mite *Oligonychus ilicis* (Acari: Tetranychidae) and *Brevipalpus phoenicis* (Acari: Tenuipalpidae) [10,26,28,49,50].

Insecticides derived from neem, *A. indica*, are particularly suitable for integrated pest management (IPM) programs due to their low contact toxicity, the need for direct ingestion, and their high selectivity towards natural enemies [29,58]. The evaluation of the repellent effect of neem NeemAzal T/S on the oviposition of *L. coffeella* in greenhouses demonstrated the potential of neem seed extract to control this pest present in coffee plantations, preventing the development of miners in the early stages of development on coffee leaves [29].

Botanical insecticides derived from orange leaves and neem oil applied to *L. coffeella* eggs reduce their larval hatching rate, demonstrating their compatibility with the survival of the predator *C. externa* [46]. The application of neem oil to *L. coffeella* eggs not only reduces larval survival but can also aid adult emergence [50]. However, a mixture of kaolin and neem oil prevented adult emergence, meaning that the addition of kaolin to neem oil delays the toxic action of neem when applied to eggs or larvae within mines, demonstrating its compatibility and ensuring the survival of natural enemies when applied in combination despite its toxic effect [52].

Variations in the efficiency of plant extracts can be attributed to the production of secondary metabolites, which is influenced by climatic conditions at the time the plants are harvested [59]. Under laboratory conditions, aqueous botanical extracts of *T. ciliata*, *T. casaretti*, *T. pallida*, *T. catigua*, *C. ambrosioides,* and *A. indica* have shown success against eggs, larvae, and pupae of *L. coffeella* [47]. Extracts of *P. lanceolata* and *M. charantia* can reduce oviposition and egg hatching in *L. coffeella*, as well as female fecundity. Both extracts contain molecules capable of influencing the sensory system of *L. coffeella* females, which are used to assess the quality of the host plant as a substrate for oviposition [60]. The ethanolic and hexanic extracts of *Dieffenbachia brasiliensis* (Veiech) (Araceae) and extracts from *Allamanda cathartica* L. (Apocynaceae) showed effectiveness in controlling *L. coffeella* after 24 h under laboratory conditions [61]. Extracts from seeds and leaves of *H. impetiginosus* have significant biopesticidal potential, are non-phytotoxic, and constitute a sustainable and environmentally sound alternative for managing *L. coffeella* preventing pupal development and completely inhibiting egg laying by adults [51].

### 4.6. Limitations and Perspectives

In recent decades, research on biological control and the use of natural products, such as plant extracts, against *L. coffeella* has progressed with the aim of integrating them into more robust and sustainable pest management systems [10]. These control methods have been much more widely studied in Brazil. However, limitations include the limited use of microbiological products, such as fungi, bacteria and other entomopathogens, the difficulty of maintaining sufficient quantities of microbials effective against *L. coffeella* for large scale use in coffee plantations, and the complexity of the population dynamics of these pests and their natural enemies. Nevertheless, the outlook is positive, with ongoing research on BCAs, as well as cultivation methods and habitat management, windbreaks, botanical pesticides and attractive plants to recruit natural enemies of the coffee leaf miner [62]. To progress efficiently and economically, it is important that research be continuously carried out to improve knowledge about *L. coffeella* and develop technological innovations, using natural and environmentally friendly approaches. Biological control of the coffee leaf miner is an important strategy for IPM, which can contribute both to reducing the use of pesticides and to minimizing the losses caused by this pest to coffee producers [63].

## 5. Conclusions

In this review, based on 28 analyzed publications, we found that BCAs are studied the most for controlling *L. coffeella* and their use has significantly progressed in recent years.

Studies on BCAs have shown that their effectiveness is largely due to the efficiency and dominance of parasitoids. The most studied entomopathogens were the fungus *M. anisopliae* and *I. fumosorosea*. Only *Metarhizium* showed high efficiency under controlled conditions.

Integrated strategies for the control *L. coffeella* aim at both pest management and environmental preservation, integrating sustainable practices. Furthermore, the results obtained with plant extracts, are considered promising and effective alternative in the IPM of *L. coffeella* given the effectiveness of some products such as neem products and orange oil.

Cover crops or intercropping promotes biological control by increasing predation and parasitism rates. Using this strategy has proved effective, since it can attract natural enemies to regulate the pest population and even create physical barriers that improve soil health. This practice, combined with other control measures, can contribute to a more sustainable and resilient production system.

## Data Availability

The original contributions presented in this study are included in the article/Supplementary Material. Further inquiries can be directed to the corresponding author.

## Supplementary Materials

The following supporting information can be downloaded at: https://www.mdpi.com/article/10.3390/insects17060636/s1. Supplementary File S1: PRISMA 2020 checklist (reference [31]); Supplementary File S2: Supplementary Information with all registered publications.
