# Supplementary material for: A Systematic Review of Biological Control Agents, Plant Extracts and Cover Crops or Intercropping for the Control of Leucoptera coffeella (Lepidoptera: Lyonetiidae)
Source: Insects. 2026 Jun 16;17(6):636. doi: 10.3390/insects17060636 (PMC13301395; doi:10.3390/insects17060636)
Supplement: Supplementary file 1 [file insects-17-00636-s001.zip › insects-4280366-File S1.pdf]

PRISMA 2020 Checklist

Section and Topic	Item #	Checklist item	Location where item is reported
**TITLE**			
Title	1	Identify the report as a systematic review.	Title
**ABSTRACT**			
Abstract	2	See the PRISMA 2020 for Abstracts checklist.	Abstract
**INTRODUCTION**			
Rationale	3	Describe the rationale for the review in the context of existing knowledge.	Introduction: There are several known methods for controlling this pest, such as chemical control, biological control through the action of entomopathogenic fungi and bacteria, predators and parasitoids, genetic control, classical breeding, semi chemicals, and botanical pesticides as another control alternative.
Objectives	4	Provide an explicit statement of the objective(s) or question(s) the review addresses.	We aimed to establish the current knowledge and limitations on the biological control of *L. coffeella* through a comprehensive systematic review, highlighting the main factors that drive the success or failure of biocontrol interventions and plant-derived botanical pesticides.
**METHODS**			
Eligibility criteria	5	Specify the inclusion and exclusion criteria for the review and how studies were grouped for the syntheses.	We considered articles that included *L. coffeella* (Lepidoptera: Lyonetidae) and publications that used biological control through the action of entomopathogenic fungi and bacteria, predators and parasitoids, and botanical pesticides.
Information sources	6	Specify all databases, registers, websites, organisations, reference lists and other sources searched or consulted to identify studies. Specify the date when each source was last searched or consulted.	On June 6, 2025, we conducted a search for scientific articles in the Web of Science, SciELO, Scopus, ScienceDirect, and other databases, as well as Google Scholar.
Search strategy	7	Present the full search strategies for all databases, registers and websites, including any filters and limits used.	This study used a systematic search in the Web of Science, SciELO, Scopus, ScienceDirect, and other databases, as well as Google Scholar, using terms such as "Coffee leaf miner". OR "*Leucoptera coffeella*" OR "biological control" OR "natural enemies" OR "parasitoids" OR "predators" OR "bacteria" OR "fungi" OR "biopesticides" OR "botanical pesticides" OR "bioactive" OR "plant extracts" OR "survey" OR "management" OR "diagnosis" OR "control" OR "alternatives control (plant extracts)" Peer-reviewed articles published between 1980 and 2025 in Portuguese, Spanish, or English were included, while books and book chapters, scientific notes editorials, dissertations and theses, and studies of characterization, identification, and selectivity of insecticides were excluded. Of the initial 130 records, 28 met the eligibility criteria.
Selection process	8	Specify the methods used to decide whether a study met the inclusion criteria of the review, including how many reviewers screened each record and each report retrieved, whether they worked independently, and if applicable, details of	For inclusion of studies, the following criteria were determined: studies with the species *L. coffeella*; articles on BCAs in the context of pest management; experiments containing at least one plant extract and/or

Section and Topic	Item #	Checklist item	Location where item is reported
		automation tools used in the process.	botanical pesticides; and studies with coffee/cover crop intercropping systems. Articles written in English, Spanish, or Portuguese, peer-reviewed, and published in journals between 1980 and 2025. Exclusion criteria included studies not focusing on control strategies, management, control alternatives, and biological control, review articles, and papers presented at conferences.
Data collection process	9	Specify the methods used to collect data from reports, including how many reviewers collected data from each report, whether they worked independently, any processes for obtaining or confirming data from study investigators, and if applicable, details of automation tools used in the process.	The searches were conducted using the terms: Coffee leaf miner. OR "*L. coffeella*" OR "biological control" OR "natural enemies" OR "parasitoids" OR "predators" OR "bacteria" OR "fungi" OR "biopesticides" OR "botanical pesticides" OR "bioactive" OR "plant extracts" OR "survey" OR "management" OR "diagnosis" OR "control" OR "alternatives control".
Data items	10a	List and define all outcomes for which data were sought. Specify whether all results that were compatible with each outcome domain in each study were sought (e.g. for all measures, time points, analyses), and if not, the methods used to decide which results to collect.	Not applicable
	10b	List and define all other variables for which data were sought (e.g. participant and intervention characteristics, funding sources). Describe any assumptions made about any missing or unclear information.	Not applicable
Study risk of bias assessment	11	Specify the methods used to assess risk of bias in the included studies, including details of the tool(s) used, how many reviewers assessed each study and whether they worked independently, and if applicable, details of automation tools used in the process.	Not applicable
Effect measures	12	Specify for each outcome the effect measure(s) (e.g. risk ratio, mean difference) used in the synthesis or presentation of results.	Not applicable
Synthesis methods	13a	Describe the processes used to decide which studies were eligible for each synthesis (e.g. tabulating the study intervention characteristics and comparing against the planned groups for each synthesis (item #5)).	Not applicable
	13b	Describe any methods required to prepare the data for presentation or synthesis, such as handling of missing summary statistics, or data conversions.	Publications eligible for the systematic review were characterized, analyzed, and presented in graphs and tables. Data on methodological approaches, control methods, and the comparison between the BCAs of *L. coffeella* were detailed. Data non temporal trend on BCAs, botanicals and cover crops or intercropping for the management of *L. coffeella*; number of publi-cations for countries on BCAs, plant extracts, and cover crops/intercropping for *L. coffeella* by country; methodological ap-proaches applied in publications on BCAs, cover crops/intercropping and plant extracts of the coffee leaf miner *L. coffeella* were detailed and the graphs were generated using Bio-Stat 5.3 software

Section and Topic	Item #	Checklist item	Location where item is reported
	13c	Describe any methods used to tabulate or visually display results of individual studies and syntheses.	We analyzed the main factors that determine the success or failure of BCA, botanicals and cover crops or intercropping interventions through field, laboratory, and semi-field performance studies. Characterization and classification of the studies in terms of % parasitism, % mortality, % predation, inoculation areas and rates, developmental stage of the leaf miner exposed, duration, and efficiency.
	13d	Describe any methods used to synthesize results and provide a rationale for the choice(s). If meta-analysis was performed, describe the model(s), method(s) to identify the presence and extent of statistical heterogeneity, and software package(s) used.	Not applicable
	13e	Describe any methods used to explore possible causes of heterogeneity among study results (e.g. subgroup analysis, meta-regression).	Not applicable
	13f	Describe any sensitivity analyses conducted to assess robustness of the synthesized results.	Not applicable
Reporting bias assessment	14	Describe any methods used to assess risk of bias due to missing results in a synthesis (arising from reporting biases).	Not applicable
Certainty assessment	15	Describe any methods used to assess certainty (or confidence) in the body of evidence for an outcome.	Not applicable
**RESULTS**			
Study selection	16a	Describe the results of the search and selection process, from the number of records identified in the search to the number of studies included in the review, ideally using a flow diagram.	Figure 1 Supplementary sheet
	16b	Cite studies that might appear to meet the inclusion criteria, but which were excluded, and explain why they were excluded.	We identified 130 publication records using the search terms. After applying the filters, 102 publications were considered unsuitable for information extraction, which allowed the selection of 28 publications eligible for systematic review analysis.
Study characteristics	17	Cite each included study and present its characteristics.	Searches were conducted for the systematic review using databases. Our strategic search resulted in 130 publications. We excluded 102 publications after applying filters and analyzed 28 titles, terms: keywords, and abstracts.
Risk of bias in studies	18	Present assessments of risk of bias for each included study.	Not applicable
Results of individual studies	19	For all outcomes, present, for each study: (a) summary statistics for each group (where appropriate) and (b) an effect estimates and its precision (e.g. confidence/credible interval), ideally using structured tables or plots.	Not applicable
Results of	20a	For each synthesis, briefly summarise the characteristics and risk of bias among	Not applicable

Section and Topic	Item #	Checklist item	Location where item is reported
syntheses		contributing studies.	
	20b	Present results of all statistical syntheses conducted. If meta-analysis was done, present for each the summary estimate and its precision (e.g. confidence/credible interval) and measures of statistical heterogeneity. If comparing groups, describe the direction of the effect.	Not applicable
	20c	Present results of all investigations of possible causes of heterogeneity among study results.	Not applicable
	20d	Present results of all sensitivity analyses conducted to assess the robustness of the synthesized results.	Table 1-4
Reporting biases	21	Present assessments of risk of bias due to missing results (arising from reporting biases) for each synthesis assessed.	Not applicable
Certainty of evidence	22	Present assessments of certainty (or confidence) in the body of evidence for each outcome assessed.	Not applicable
**DISCUSSION**			
Discussion	23a	Provide a general interpretation of the results in the context of other evidence.	This systematic review highlights the current knowledge and limitations of biological control of *L. coffeella* through a comprehensive systematic literature review, emphasizing the main factors that determine the success or failure of biocontrol interventions and plant-derived botanical pesticides. Most of the research is conducted in Brazil and Porto Rico. Ecological strategies for the control of *L. coffeella* demonstrate the importance of parasitoids and predators in the field.
	23b	Discuss any limitations of the evidence included in the review.	The main limitations of the review include the restricted use of microbiological products, such as fungi and bacteria; the difficulty in maintaining sufficient quantities of antimicrobials effective against *L. coffeella* for large-scale use in coffee plantations; and the complexity of the population dynamics of these pests and their natural enemies.
	23c	Discuss any limitations of the review processes used.	In recent decades, research on biological control and the use of natural products, such as plant extracts, against *L. coffeella* has progressed with the aim of integrating them into more robust and sustainable pest management systems. These control methods have been much more widely adopted in Brazil.
	23d	Discuss implications of the results for practice, policy, and future research.	To progress efficiently and economically, it is important that research is continuously carried out to improve knowledge about *L. coffeella* and technological innovations, using natural and environmentally friendly approaches. Biological control of the coffee leaf miners is an important strategy for IPM, which can contribute both to reducing the use of pesticides and to minimizing the losses caused by this pest to coffee

PRISMA 2020 Checklist

Section and Topic	Item #	Checklist item	Location where item is reported
			producers.
**OTHER INFORMATION**			
Registration and protocol	24a	Provide registration information for the review, including register name and registration number, or state that the review was not registered.	This review was not registered in any systematic review Registry.
	24b	Indicate where the review protocol can be accessed, or state that a protocol was not prepared.	This review was not registered in any systematic review Registry.
	24c	Describe and explain any amendments to information provided at registration or in the protocol.	This review was not registered in any systematic review Registry.
Support	25	Describe sources of financial or non-financial support for the review, and the role of the funders or sponsors in the review.	This work was financially supported by the State University of Southwest Bahia (UESB) and Council of Technological and Scientific Development (CNPq).
Competing interests	26	Declare any competing interests of review authors.	Conflicts of interest<br>None.
Availability of data, code and other materials	27	Report which of the following are publicly available and where they can be found: template data collection forms; data extracted from included studies; data used for all analyses; analytic code; any other materials used in the review.	Supplementary sheet containing the complete data extracted in the systematic review process.

From: Page MJ, McKenzie JE, Bossuyt PM, Boutron I, Hoffmann TC, Mulrow CD, et al. The PRISMA 2020 statement: an updated guideline for reporting systematic reviews. BMJ 2021; 372: n71. doi: 10.1136/bmj.n71. This work is licensed under CC BY 4.0. To view a copy of this license, visit https://creativecommons.org/licenses/by/4.0/ -----
